# Evaluate the effect of coarse aggregates on cement hydration heat and concrete temperature modelling using isothermal calorimetry

**DOI:** 10.1016/j.heliyon.2024.e38322

**Published:** 2024-09-26

**Authors:** Yaowen Tan, Kangkang Tang

**Affiliations:** Department of Civil and Environmental Engineering, Brunel University London, Uxbridge, Middlesex, UB8 3PH, UK

**Keywords:** Cement hydration heat, Coarse aggregate, Isothermal calorimetry, Semi-adiabatic calorimetry, Finite element modelling

## Abstract

The early-age temperature rise in concrete, induced by cement hydration, poses a significant risk of thermal cracking. Accurate prediction of concrete hydration temperature is essential for thermal cracking prevention. Cement hydration heat obtained from isothermal calorimetry has been applied to concrete temperature modelling by previous studies. Isothermal calorimetry often excludes coarse aggregates due to the calorimeter capacity limitations, assuming mortar hydration heat can represent concrete, which may neglect the hydration delay effect of coarse aggregates. This study uses an isothermal calorimeter capable of accommodating coarse aggregates to measure the hydration heat of concrete and equivalent mortar, evaluating the validity of this assumption. Results show that the 3-day cumulative hydration heat of concrete exceeds that of mortar, especially at elevated curing temperatures. Significant differences were found in the activation energy and hydration parameters between concrete and mortar, indicating that the presence of coarse aggregates affects samples’ temperature sensitivity and hydration heat development. Concrete temperature finite element modelling, validated by semi-adiabatic calorimetry, demonstrates that models based on concrete isothermal calorimetry data provide higher accuracy than those based on mortars. This study demonstrates that the hydration heat development, activation energy, and hydration parameters differ significantly between mortar and concrete. Concrete temperature models based on mortar hydration heat data can result in prediction errors exceeding 5 %. This study recommended employing micro-concrete samples in isothermal calorimetry to replicate actual concrete mixes.

## Graphical abstract

**Figure:**
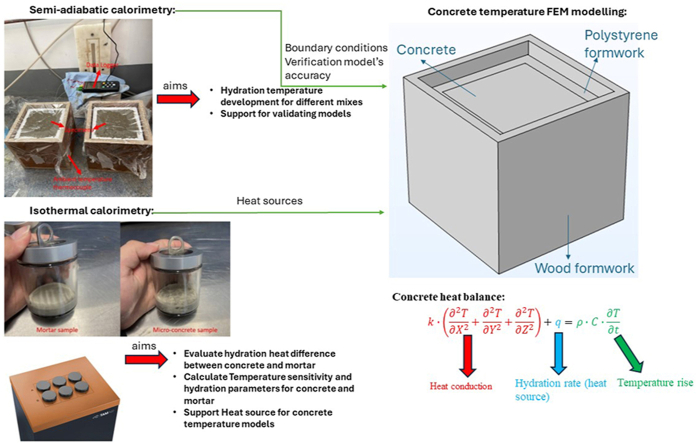

## 1 Introduction

The early-age temperature development of concrete is a crucial factor affecting the integrity and safety of concrete structures. During the initial hydration phase, the exothermic reactions of cement release substantial heat, causing an increase in the internal temperature of the concrete. This temperature increment induces expansion, followed by contraction as the concrete cools after reaching peak temperatures. When this thermal contraction is restrained by adjacent structures or temperature gradients, tensile stresses develop. At early ages, concrete has not yet attained sufficient maturity and tensile strength, these stresses may exceed the material's capacity, resulting in early-age thermal cracking [[1], [2], [3], [4], [5]]. Such thermal cracks provide pathways for erosive substances like water, chlorides, and sulphates, accelerating the degradation process and compromising the durability and lifespan of concrete structures [6]. To mitigate the risk of early-age thermal cracking, various strategies are employed in construction, including embedding water-cooling pipes [7,8], concrete expansion joints [[9], [10], [11]], and replacing part of cement with supplementary cementitious materials (SCMs) such as ground granulated blast furnace slag (GGBS), which has a lower heat of hydration than ordinary Portland cement [1,[12], [13], [14]]. Accurate prediction of the early-age hydration temperature development in concrete is crucial for forecasting thermal cracking and implementing effective mitigation measures.

Calorimetry is commonly employed in the laboratory to obtain the hydration heat of cement and the temperature evolution of specimens, encompassing adiabatic, semi-adiabatic, and isothermal calorimetry. The adiabatic calorimetry tests are conducted with specimens controlled by a temperature control system that synchronizes the surrounding temperature with the specimen's temperature, thereby minimizing heat loss and approximating “adiabatic” conditions [[15], [16], [17]]. This method can be utilized to simulate the temperature development at the core of mass concrete structures [18,19]. In contrast, the semi-adiabatic calorimetry test, while similar to the adiabatic method, allows for some heat loss and does not require a temperature control system [16,20,21]. Instead, specimens are typically cured under uncontrolled ambient conditions. Thus, semi-adiabatic calorimetry is an effective laboratory method for simulating the temperature development of in-situ concrete [1,12,22]. Both adiabatic and semi-adiabatic calorimetry directly measure the specimen's temperature, necessitating a back-calculation of hydration heat based on the specific heat capacity of the specimen.

In contrast, isothermal calorimetry directly measures the hydration heat power at a constant temperature, eliminating the need to know the specific heat capacity of the material. Modern isothermal calorimeters, such as the TAM Air [23], offer a wide operational temperature range from 5 °C to 90 °C. This method typically requires minimal mortar specimens (tens of grams), making it a cost-effective approach in terms of raw material preparation. By conducting isothermal calorimetry tests at various temperatures, the influence of temperature on hydration reactions can be evaluated. By maintaining a constant temperature, isothermal calorimetry avoids the confounding effects of temperature on the hydration process, thereby elucidating the impact of other variables, such as mix composition or additives, on the hydration process [18,24,25].

The calorimetry data obtained from these experiments can be instrumental in simulating the temperature development of concrete via the concrete maturity method. The most widely used concrete maturity formula is the concept of equivalent age (Eq. (2)) based on the Arrhenius equation (Eq. (1)). The fundamental assumption of the maturity method is that concrete with the same mix and maturity has approximately the same strength, regardless of temperature and time histories [26,27]. In the context of predicting concrete hydration temperature, this assumption extends to concrete with the same mix and maturity will also exhibit the same degree of hydration and cumulative hydration heat [28]. Therefore, knowing the temperature and age of the concrete specimen at a specific time step allows for the estimation of its degree of hydration and cumulative hydration heat based on calorimetry data. Subsequently, using the material's specific heat capacity, the temperature for the next time step can be calculated. This iterative process theoretically enables the prediction of the entire temperature development during the hydration process, provided the initial temperature and specific heat capacity of the concrete specimen are known.

(1) $$Q\left(T\right)={Ae}^{\left(\frac{-E_{a}}{R\cdot T}\right)}$$

(2) $$\begin{array}{c} t_{e}=\sum e^{\frac{-E_{a}}{R}\left(\frac{1}{T}-\frac{1}{T_{r}}\right)}\cdot \Delta t \end{array}$$

Where Q(T) is the cement hydration flow at temperature T (W/g), A is a constant of proportionality, R is the universal gas constant = 8.314 (J/Kmol), T is the concrete temperature (K), t_e_ is the concrete equivalent age (h), T_r_ is the reference temperature (K), E_a_ is the activation energy (J/mol). E_a_ refers to the minimum energy required for a chemical reaction to occur. A higher activation energy indicates greater sensitivity to temperature changes. E_a_ can be determined through mortar strength tests at different temperatures or isothermal calorimetry tests conducted at different temperatures. This method's principle involves a straightforward mathematical transformation of the Arrhenius equation, as shown in Eq. (3). This transformation elucidates the direct linear relationship between the reciprocal of temperature (1/T) and the natural logarithm of the maximum hydration rate (ln(Q_max_)), with the slope of the resulting linear fit being equal to E_a_/R. This approach is recommended by ASTM C1074 [29] and several studies [28,30,31].

(3) $$\begin{array}{c} \ln\left(Q_{\max}\right)=-\frac{E_{a}}{RT}+lnA \end{array}$$

The actual prediction process is often more complex. For instance, the continuously fluctuating ambient temperature of in-situ concrete affects its heat dissipation, subsequently influencing the internal temperature distribution and hydration heat development [1,22,32,33]. This significantly increases the computational load. Such issues can be effectively addressed using the Finite Element Method (FEM). Modern commercial FEM software can incorporate these external factors into the boundary conditions and efficiently compute the hydration heat and temperature development at each time step. Numerous studies have developed effective models for predicting concrete temperature evolution. For example, Jedrzejewska et al. [34] evaluated the accuracy of different hydration heat equations on concrete temperature prediction results through FEM modelling; Lim et al. [16] and Xie et al. [35]used semi-adiabatic calorimetry tests within FEM models to predict the adiabatic temperature rise of concrete; Liu et al. [36] and Al-Hasani et al. [18] utilized isothermal calorimetry tests within FEM models to predict the temperature development of concrete structures.

Xu et al. [37] assessed the accuracy of different calorimetry tests in characterizing cement hydration properties and predicting concrete temperature development. They obtained hydration heat evolution data from both semi-adiabatic and isothermal calorimetry tests. These results were subsequently incorporated into their model to simulate the temperature development within concrete pavements. The results indicated that simulations based on semi-adiabatic calorimetry provided better hydration degree and temperature predictions than those based on isothermal calorimetry. Xu et al. [37] attributed this discrepancy to the absence of coarse aggregates in the mortar samples used in isothermal calorimetry, which led to an oversight of the “delay effect” by coarse aggregates. This potential error highlights a limitation of isothermal calorimetry in concrete research, where the limited capacity of the ampoules or channels, such as the 20 mL capacity of the widely used 8-channel TAM Air calorimeter, prevents the inclusion of coarse aggregates. Therefore, Xu et al.’s speculation about these errors was not further substantiated. Many studies [17,18,36] use equivalent mortars or pastes with the same binder content and water-to-cement ratio as concrete for isothermal calorimetry tests, assuming that the hydration heat development of these mortars or pastes can represent concrete. Wadsö [38], using an 8-channel TAM Air isothermal calorimeter, investigated the effect of adding fine aggregates with a particle size of 5 mm on the cement hydration heat development and found that samples containing aggregates had a higher peak rate of heat release compared to pure cement paste. From Wadsö’s provided charts, it can be inferred that the difference in peak heat release rate was about 0.3 mW/g. Based on this slight difference, Wadsö considered that the hydration curves of cement paste and mortar could be equivalent. Wadsö further assumed that the isothermal calorimetry results of equivalent mortars or pastes could represent the hydration heat of concrete. However, Wadsö also recognized the limitation of channel capacity, which prevented larger-sized coarse aggregates commonly used in actual concrete mixtures from being included, thus preventing further substantiation of this equivalence been extended to concrete.

Therefore, whether the hydration heat development of equivalent mortars can substitute for concrete remains an unproven and controversial issue. Applying the hydration heat of mortars to predict temperature development in concrete may lead to decreased prediction accuracy due to the neglect of coarse aggregates in concrete. This study aims to address this gap by utilizing an isothermal calorimeter capable of accommodating coarse aggregates to compare the hydration heat development, apparent activation energy, and hydration parameters of mortar and concrete samples. These comparisons will evaluate the validity of substituting the hydration heat of mortar for that of concrete. Furthermore, the study has developed a concrete temperature FEM model, with heat sources derived from the isothermal calorimetry results of both mortar and concrete samples. The accuracy of these simulations will be used to assess the potential errors in predicting concrete temperature development when using the hydration heat of mortar. Additionally, this research will also explore the effect of substituting a portion of cement with GGBS on mitigating early-age hydration heat development in concrete.

## 2 Experimental procedures

### 2.1 Raw materials

The cement used in this research is CEM-I 52.5N, a high-strength cement produced by CEMEX UK Cement Ltd. This cement conforms to the standards outlined in BS EN 197–1:2011 [39]. This research employs Ground Granulated Blast-Furnace Slag (GGBS) as a partial replacement for cement to investigate its suppressive effect on cement hydration heat and the early-age hydration temperature rise in concrete. The GGBS used in this research is TEES SF REGEN GGBS, produced by Hanson UK. This GGBS complies with the standards of BS EN 15167–1:2006 [40]. The oxide composition of the cement and GGBS were determined through X-ray fluorescence (XRF) testing, with the results presented in Table 1. The contents of the four primary cement minerals (C_3_S, C_2_S, C_3_A, and C_4_AF) were determined through Bogue calculations based on the oxide compositions (Eqs. (4), (5), (6), (7)). The results of these calculations are presented in Table 1.

(4) $$\begin{array}{c} p_{C_{3}S}=4.071p_{CaO}-7.600p_{SiO_{2}}-6.718p_{{Al}_{2}O_{3}}-1.430p_{{Fe}_{2}O_{3}} \end{array}$$

(5) $$\begin{array}{c} p_{C_{2}S}=2.867p_{SiO_{2}}-0.7544p_{C_{3}S} \end{array}$$

(6) $$\begin{array}{c} p_{C_{3}A}=2.650p_{{Al}_{2}O_{3}}-1.692p_{{Fe}_{2}O_{3}} \end{array}$$

(7) $$\begin{array}{c} p_{C_{4}AF}=3.043p_{{Fe}_{2}O_{3}} \end{array}$$

Where P_i_ represents the percentage of component i in the total cement (by weight).

**Table 1:** Oxide Compound of cement and GGBS.

Oxide Compound	Content (%)	
	CEM-I 52.5N	GGBS
CaO	58.70	35.20
SiO_2_	21.32	35.49
Al_2_O_3_	3.83	9.69
Fe_2_O_3_	2.95	0.92
K_2_O	0.42	0.35
MgO	2.32	4.62
SO_3_	4.25	1.87
P_2_O_5_	1.50	1.45
TiO_2_	0.23	0.68
SrO	0.11	0.05
MnO	0.07	0.29
Mineral Compound (Bogue)		
C_3_S	46.99	–
C_2_S	25.70	–
C_3_A	5.16	–
C_4_AF	8.98	–

The coarse aggregate employed in this study is locally sourced crushed riverbed gravel. Adhering to the procedures outlined in BS EN 933–1:2012 [41] and BS EN 12620: 2013 [42], the coarse aggregate was sieved using a sieving machine to obtain a uniform grading of 10 mm. This study employed medium sand as the fine aggregate, with a typical size range of 0.25–0.5 mm, as defined in BS EN 12620:2013 [42]. The density (Saturated Surface Dry (SSD) Specific Gravity and Specific Gravity) and water absorption rate of coarse and fine aggregates were determined following the testing steps specified in AASHTO T 85 [43] and AASHTO T 84 [44] separately, with the results detailed in Table 2.

**Table 2:** Density and water absorption of aggregates.

	SSD specific gravity (kg/m^3^)	Water absorption (%)	Specific gravity (kg/m^3^)
Coarse aggregate (10 mm gravel)	2550	0.78	2530
Fine aggregate (medium sand)	2476	1.86	2430

### 2.2 Concrete semi-adiabatic calorimetry test

The purpose of the semi-adiabatic calorimetry test is to simulate the early-age hydration temperature development of in-situ concrete. To investigate the impact of GGBS on early-age hydration temperature development, semi-adiabatic calorimetry tests were conducted on concrete mixes where GGBS replaced 30 % and 50 % of CEM I by weight. The compositions of these mixes are detailed in Table 3.

**Table 3:** Semi-adiabatic concrete mixes.

	CEM I (kg/m^3^)	GGBS (kg/m^3^)	Water (kg/m^3^)	Coarse aggregate (kg/m^3^)	Fine aggregate (kg/m^3^)
0 % GGBS concrete	450	0	246	1017	696
30 % GGBS concrete	315	135	246	1017	696
50 % GGBS concrete	225	225	246	1017	696

Concrete specimens were prepared using a 160-L batch mixer. Aggregates were pre-dried in an oven for 24 h and cooled to room temperature before mixing. The fresh concrete was then cast into specially designed moulds made of expanded polystyrene and timber boards. The expanded polystyrene had a thickness of 2 cm on the four sides and 3 cm at the base, with an internal cavity measuring 15 cm in length, width, and height, and the outer length and width are 19 cm, and the height is 18 cm. The dimensions of the outer timber board were slightly larger, with a thickness of 1.5 cm and internal dimensions of 20 cm in length, width, and height, resulting in external dimensions of 23 cm in length and width and 21.5 cm in height. The expanded polystyrene was placed within the timber board, and the concrete was poured into the expanded polystyrene, leaving the concrete's top surface in direct contact with the air. The top surface of the concrete specimens was immediately covered with a thin layer of cling film after casting to prevent moisture loss. The cling film is considered as no thermal insulation.

**Fig. 1:**
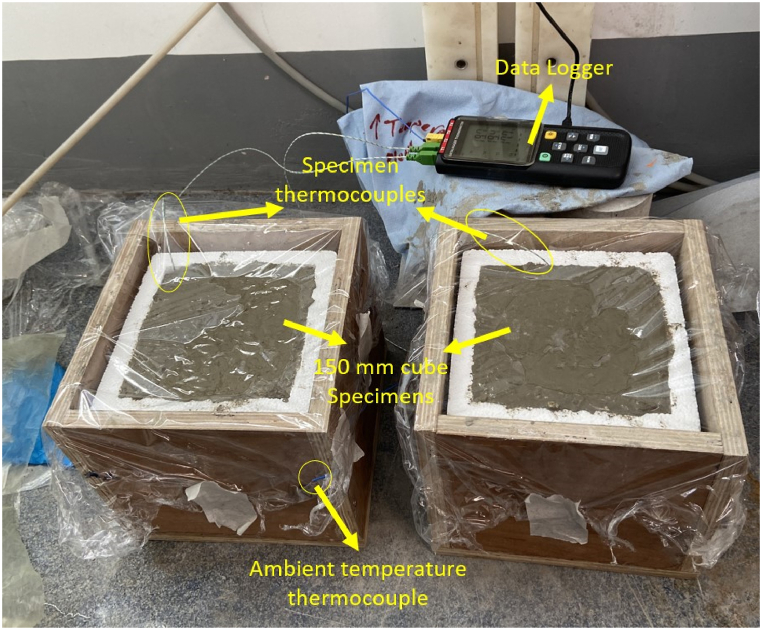
Semi-adiabatic calorimetry test setup.

During casting, type-K thermocouples were embedded at the core of each specimen to monitor temperature development, connected to a 4-channel temperature data logger with a precision of 0.1 °C for recording. After casting, the specimens were placed in an air-conditioned laboratory environment for curing. It is important to note that, unlike an environmental chamber that can maintain a nearly constant temperature, the air conditioning system keeps the room temperature within a relatively narrow range of fluctuations. This fluctuating temperature environment is intended to simulate the typical variable temperature conditions experienced by in-situ concrete. The ambient temperature was also recorded using an additional thermocouple. For each mix, two specimens were tested, and temperature data were logged at 10-min intervals over 72 h. Fig. 1 shows a photograph of the semi-adiabatic calorimetry test setup.

### 2.3 Isothermal calorimetry test

Isothermal calorimetry tests were conducted to measure the hydration heat development of samples with varying GGBS contents at different temperatures. A primary objective of this study was to assess the influence of coarse aggregates on the hydration heat development of the samples. To accommodate the 10 mm coarse aggregates utilized in this study, a three-channel TAM Air isothermal calorimeter, equipped with ampoules of 125 mL volume, was employed. This calorimeter has an accuracy of ±8 μW for the heat flow sensor and an accuracy of ±0.02 °C for the thermostat.

**Fig. 2:**
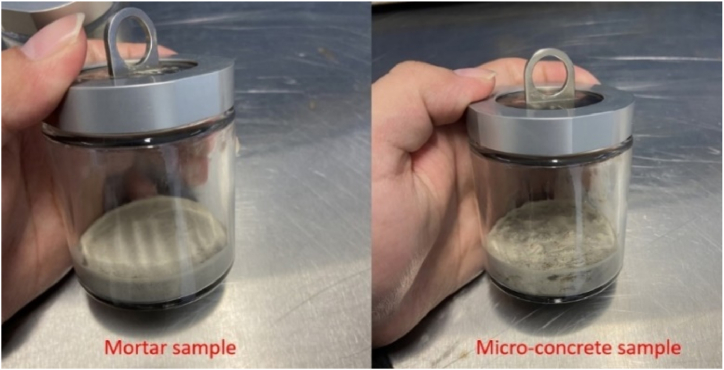
Hardened mortar sample and micro-concrete sample entirely concentrated at the ampoule's base.

The concrete mix for these tests was identical to that of the semi-adiabatic calorimetry, in which the GGBS was used to replace 30 % and 50 % of CEM I separately (Table 3) to investigate the impact of GGBS on hydration heat development. Each concrete sample, referred to as a “micro-concrete sample”, weighed 48.18 g. Additionally, equivalent mortar samples were also tested simultaneously to investigate the effect of coarse aggregates on the development of hydration heat (as shown in Fig. 2). The equivalent mortar samples were prepared following the definition of equivalent mortar in ASTM C1074 [29]: the fine aggregate-to-cement ratio (by weight) in the equivalent mortar equated to the coarse aggregate-to-cement ratio in the concrete. This allows the equivalent mortar to exhibit similar hydration heat development to concrete. The specific mix details of the isothermal calorimetry are provided in Table 4 (the binder consists of both cement and GGBS). Each sample was tested at four different temperatures: 20 °C, 30 °C, 40 °C, and 50 °C to evaluate the influence of temperature on hydration heat development and provide thermal source data for FEM model development (which will be discussed in detail in Section 3).

**Table 4:** Isothermal calorimetry sample mixes and reference sample.

	Binder (g)	Deionized water (g)	Coarse aggregate (g)	Fine aggregate (g)	Total weight (g)	Reference sample (sand) (g)
Mortar	9	4.92	0	20.34	34.26	48.50
Micro-concrete	9	4.92	20.34	13.92	48.18	62.40

## 3 Finite element modelling

### 3.1 Concrete heat balance and heat loss

In the FEM modelling process, the development of concrete temperature is simulated through a commercial finite element software, COMSOL Multiphysics 6.1 [45]. The heat balance inside the concrete can be expressed by a three-dimensional heat diffusion equation based on Fourier heat transfer principle:

(8) $$\begin{array}{c} k\cdot \left(\frac{\partial^{2}T}{\partial X^{2}}+\frac{\partial^{2}T}{\partial Y^{2}}+\frac{\partial^{2}T}{\partial Z^{2}}\right)+Q=\rho \cdot C_{p}\cdot \frac{\partial T}{\partial t} \end{array}$$

Where Q is the heat generation rate of the cement (W/m^3^), X, Y, Z represent three different axes in the concrete, T is the concrete temperature (°C), ρ is the density of the concrete (kg/m^3^), and C_p_ is the heat capacity of concrete (J/kg·K).

The concrete heat loss is defined in this model via thermal convection (as shown in Eq. (9)), which is primarily dependent on the temperature differential between the concrete surface (or insulation layer surfaces) and the surrounding air, as well as the convection coefficient, h_c_. The value of h_c_ is chiefly influenced by wind velocity. Due to the absence of wind velocity data within the laboratory setting, the convection coefficient between the concrete surface and air is adopted from the literature [6], with a value of 5.6 W/m^2^·°C. The ambient (curing) temperature during the concrete semi-adiabatic curing was monitored and will be inputted into the model as the boundary condition to govern the concrete heat loss.

(9) $$\begin{array}{c} q_{c}=h_{c}\cdot \left(T_{s}-T_{a}\right) \end{array}$$

Where q_c_ is the heat flux at the concrete or insulation layer surfaces (W/m^2^), h_c_ is the convection coefficient, which represents the heat transfer capacity between solids and air (W/m^2^ ·°C), T_s_ is the temperature of the concrete or insulation layer surfaces (°C), and T_a_ is the ambient (air) temperature (°C).

It is imperative to note that only the top surface of the concrete specimen is directly exposed to the surrounding environment, while the four sides and the bottom are encapsulated by two layers of insulation materials, namely polystyrene and timber board, as depicted in Fig. 1. Consequently, the convective coefficient, h_c_ (5.6 W/m^2^·°C), applies solely to the thermal exchange between the concrete's top surface and the air.

The thermal convection from the insulation layer surfaces was defined through an equivalent convection coefficient h_eq_ given by the following formula [28,46]:

(10) $$\begin{array}{c} h_{eq}={\left(\frac{1}{h_{c}}+\sum_{1}^{n}\frac{L_{i}}{k_{i}}\right)}^{-1} \end{array}$$

Where h_eq_ is the equivalent convection coefficient (W/m^2^ ·°C), h_c_ is the convection coefficient between the concrete surface and air (W/m^2^ ·°C), L_i_ is the i-th insulation layer's thickness (mm), and k_i_ is the i-th insulation layer's thermal conductivity (W/m·°C).

The thermal conductivities of polystyrene and timber board were derived from literature [6,47]. Table 5 presents the thermal properties of the two insulating materials. The calculated value of the equivalent convection coefficient, h_eq_, for the sides and bottom of the specimen, are 1.47 and 1.14 W/m·°C, separately.

**Table 5:** Thermal properties of hardened concrete and insulation materials.

	Thermal conductivity (W/m·°C)	Specific heat capacity (J/kg·°C)
Polystyrene	0.0624	1040
Timber board	0.15	122

### 3.2 Definition of heat source

In the heat transfer model of concrete, the only internal heat source stems from the hydration heat released during the hydration reaction between cementitious materials (CEM I and GGBS in this study) and water.

The development of heat of hydration in cement can be quantified through the degree of hydration α. In isothermal calorimetry tests, the degree of hydration is determined by the ratio of the heat released by the sample to the maximum potential heat of hydration (refer to Eq. (11)). Consequently, the degree of hydration ranges from 0 to 1, with 1 representing the degree of hydration after complete cement hydration, a state that is virtually unattainable in practice.

(11) $$\begin{array}{c} \alpha \left(t\right)=\frac{H\;\left(t\right)}{H_{u}} \end{array}$$

Where H(t) is the cumulative hydration at time t, H_u_ is the theoretical maximum heat of hydration of the cement.

The ultimate heat of hydration of the cement (H_cem_) was calculated using the formula proposed by Schindler and Folliard [48] (Eq. (12)), which is based on the mineral composition of the cement. Schindler and Folliard [48] also suggest that the reduction in ultimate heat of hydration (H_u_) due to the partial replacement of cement with GGBS can be considered proportional to the replacement level, as expressed in Eq. (13).

(12) $$H_{cem}=500p_{C_{3}S}+260p_{C_{2}S}+866p_{C_{3}A}+420p_{C_{4}AF}+624p_{{so}_{3}}+1186p_{Free\;lime}+850p_{MgO}$$

(13) $$\begin{array}{c} H_{u}=H_{cem}\cdot p_{cem}+461p_{slag}+1800p_{FA-CaO}\cdot p_{FA} \end{array}$$

Where p_i_ represents the percentage of component i in the total cement (by weight), p_slag_ is the percentage of slag in the total cementitious materials (by weight), p_FA_ is the percentage of FA in the total cementitious materials (by weight), p_FA-CaO_ is the percentage of mass of CaO in fly ash to total mass of fly ash.

In this model, the degree of hydration α is described using the well-known Three Parameter Equation (Danish model) proposed by Freiesleben Hansen and Pedersen [49], as shown in Eq. (14). This model was originally developed to capture the development of cement hydration heat under isothermal conditions. However, when applied to non-isothermal scenarios, such as the temperature modelling of in-situ concrete in this study, the continuously changing internal temperature of the concrete complicates the evolution of the degree of hydration over time, making it difficult to represent with a simple equation. Therefore, in this study, the time variable in Eq. (14) is expressed as the equivalent age rather than the actual age to account for the effects of varying concrete temperatures. The model requires the apparent activation energy (E_a_) to calculate the equivalent age t_e_. E_a_ is determined based on the results of isothermal calorimetry, as described in Eqs. (1), (3) in Section 1. The detailed results of the E_a_ calculation are provided in Section 4.3.

(14) $$\begin{array}{c} \alpha \left(t_{e}\right)=\alpha_{u}\cdot e^{\left(-{\left(\frac{\tau }{t_{e}}\right)}^{\beta }\right)} \end{array}$$

Where α(t_e_) is the hydration degree at equivalent age t_e_ of concrete. The parameters α_u_, τ, and β are hydration parameters of cement hydration. α_u_ is the ultimate hydration degree that the hydration reaction can be attend, which is a parameter specific to the material, a higher α_u_ leads to a higher final degree of hydration, and more total heat is available for hydration. τ is the hydration heat time parameter, a higher value of τ indicates a more pronounced delay in the hydration process. β is the hydration heat development shape parameter, which characterizes the shape of the hydration-time curve, predominantly dictating the gradient of its primary linear segment. An increased β value suggests a more rapid hydration rate during the early-age [37,50].

The isothermal calorimetry test results will be employed to determine the hydration degree at each time point (t,α) via Eq. (11), which correlates cumulative heat release with hydration degree. These time-based hydration degrees are then transformed into the equivalent age format (t_e_,α) via Eq. (2), which accounts for temperature variations throughout the hydration process. The equivalent age-based hydration degree data (t_e_,α) are subsequently applied in Eq. (14) to derive the hydration parameters α_u_, τ, and β through non-linear regression analysis. This regression analysis is performed by MATLAB's Curve Fitting Toolbox. These hydration parameters are essential for accurately modelling hydration kinetics and the corresponding thermal evolution in concrete. Detailed results are presented in Section 4.3.

The Domain Ordinary Differential Equations (DODE) physics interface within COMSOL Multiphysics will be utilized to define the equivalent age t_e_, degree of hydration α, and cumulative hydration heat H for each time step. The hydration heat release rate Q at each timestep will be determined based on these DODE equations.

The first DODE will initially articulate the concrete's equivalent age t_e_. This process will translate the age t of concrete at a specific temperature to the equivalent age t_e_ of the same concrete mix at a reference temperature set at 20 °C of this study. The equivalent age equation is reformulated into a differential equation as follows:

(15) $$\begin{array}{c} \frac{dt_{e}}{dt}=e^{\frac{-E_{a}}{R}\left(\frac{1}{T}-\frac{1}{T_{r}}\right)} \end{array}$$

The second DODE will be employed to express the hydration degree α of concrete for a given time step. By converting Eq. (14) into a form of an ordinary differential equation, the following formal can be obtained:

(16) $$\begin{array}{c} \frac{d\alpha \left(t\right)}{dt}=\frac{d\alpha \left(te\right)}{dt_{e}}\cdot \frac{dt_{e}}{dt}=\alpha_{u}\cdot e^{\left(-{\left(\frac{\tau }{t_{e}}\right)}^{\beta }\right)}\cdot {\left(\frac{\tau }{t_{e}}\right)}^{\beta }\cdot \left(\frac{\beta }{t_{e}}\right)\cdot \frac{dt_{e}}{dt} \end{array}$$

The third DODE is formulated to express the concrete's cumulative hydration heat, H, at a given time step. The value of cumulative hydration heat at a certain degree of hydration can be derived from the definition of hydration degree (Eq. (11)). Consequently, the differential equation representing the cumulative hydration heat, H, with respect to actual age, t, can be articulated as follows:

(17) $$\begin{array}{c} \frac{dH\left(t\right)}{dt}=H_{u}\cdot \frac{d\alpha \left(t\right)}{dt} \end{array}$$

Eqs. (15), (16), (17) represent the three distinct ODEs defined within the model to characterize the heat source. The heat release rate Q equates to the time derivative of the cumulative hydration heat:

(18) $$\begin{array}{c} Q\left(t\right)=C_{b}\cdot \frac{dH\left(t\right)}{dt} \end{array}$$

Where Q(t) is the hydration heat release rate at an actual age t (J/m^3^), C_b_ is the binder content of concrete (g/m^3^).

The isothermal calorimetry test results for micro-concrete and equivalent mortar samples will be employed to compute the hydration parameters in Eq. (14) through nonlinear fitting. The calculated hydration parameters will be input into the DODEs of the FEM model. The DODE results will be utilized to calculate the heat source at each time step within the model. Finally, the impact of using mortar hydration heat as a substitute for concrete on the prediction of concrete temperature will be evaluated by comparing the results obtained from the two heat sources (derived from micro-concrete or equivalent mortar).

## 4 Results and discussion

### 4.1 Concrete semi-adiabatic calorimetry test results

**Fig. 3:**
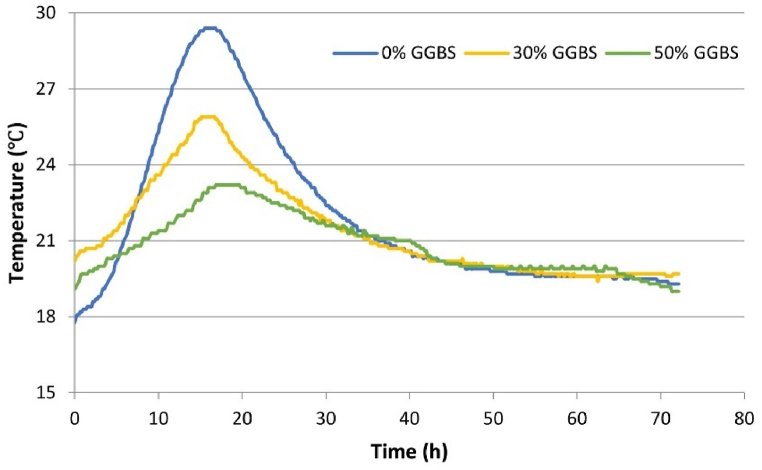
Concrete semi-adiabatic temperature results.

**Fig. 4:**
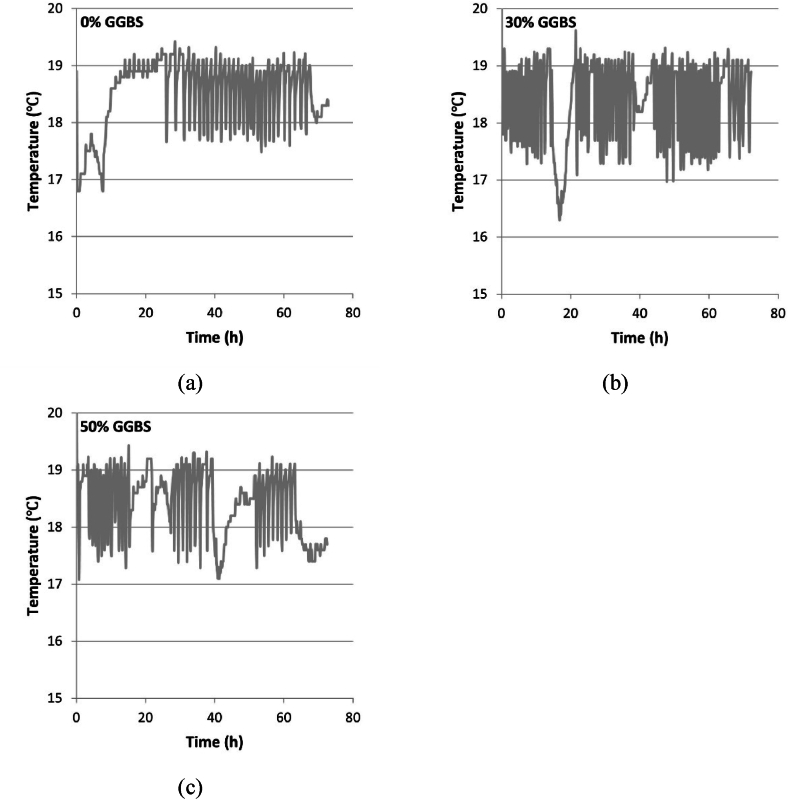
Curing temperatures of specimens: (a) 0 % GGBS; (b) 30 % GGBS; (c) 50 % GGBS.

In the semi-adiabatic calorimetry tests, GGBS was used to replace 30 % and 50 % of CEM I to investigate the effect of GGBS on mitigating the hydration temperature of concrete. The core temperature of the concrete specimens and the ambient (curing) temperature within the laboratory were monitored. The temperature profiles of concrete specimens with varying GGBS contents are presented in Fig. 3. Fig. 4 depicts the curing temperature conditions during the experiments.

The temperature development curves of concrete with varying GGBS contents reveal a clear trend. As the ratio of GGBS substituting for CEM I increases, the peak temperature of the concrete correspondingly decreases (as illustrated in Fig. 3). Substituting 50 % of CEM I with GGBS reduces the peak temperature by approximately 21.1 %. However, the time taken to reach the peak temperature does not have a direct and noticeable correlation with the GGBS content. This observation can be attributed to the variable temperature environment where the specimens were placed, as presented in Fig. 4. For example, in Fig. 4 (b), the compact, small-range fluctuations (such as 0–15 h, 22–38 h) are attributed to the normal operational variations of the air conditioning system. While the system is nominally intended to maintain a constant temperature of 20 °C for the entire laboratory, the actual temperature control range is limited to the vicinity of the air conditioner's built-in thermostat. Since the specimens are placed at a certain distance from the air conditioner to prevent direct airflow, it takes time for temperature changes around the specimens to be detected by the air conditioner. The system only adjusts its operational power after detecting these fluctuations to restore the environment to the set point, leading to ongoing small-scale, real-time temperature fluctuations around the specimens. The observed larger fluctuations (such as 15–22 h) may result from significant temperature changes due to personnel entering and exiting the laboratory. The casting temperatures, initial temperatures, and curing temperatures of different specimens varied among mixes, influenced by the weather conditions on the day of the experiment. Although there were slight differences in ambient temperatures across specimens, the variations were minimal. However, the initial temperatures showed more significant discrepancies (Fig. 3).

**Fig. 5:**
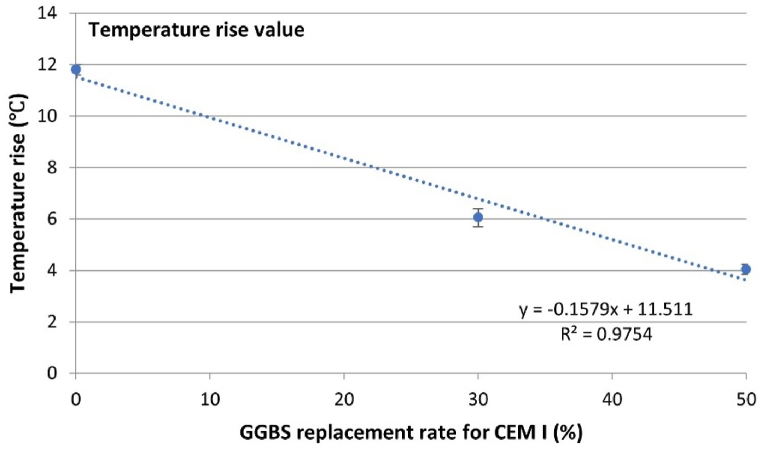
Variation of temperature rise value with GGBS content.

To facilitate a clearer understanding of the influence of GGBS content on the hydration temperature of concrete, this study introduces two additional indicators: “Temperature rise value (T_r_)”. to elucidate the impact of GGBS addition on concrete temperature development. The “Temperature rise value (T_r_)” is defined as the difference between the initial and peak temperatures of the concrete (as shown in Eq. (19)), representing the maximum temperature increase during the hydration process. The calculation results of Tr demonstrate a linear correlation with the GGBS content, as depicted in Fig. 5. The findings indicate that substituting 50 % of CEM I with GGBS reduces approximately 63.4 % in the maximum temperature rise value. This significant reduction in T_r_ underscores the effective temperature control afforded by GGBS, offering promising prospects for mitigating the risk of thermal cracking in concrete structures.

(19) $$\begin{array}{c} T_{r}=T_{ini}-T_{peak} \end{array}$$

Where T_r_ is the maximum temperature increase during the hydration process (°C), T_ini_ is the initial temperature of the concrete specimen (°C), T_peak_ is the peak temperature of the concrete specimen (°C).

Table 6 summarizes key indicators during the temperature development process for each specimen, including initial temperature, peak temperature, time to reach peak temperature, and the maximum temperature increase T_r_. It was observed that the 0 % GGBS concrete had the lowest initial temperature but the highest peak temperature. Conversely, the 30 % GGBS concrete, despite having the highest initial temperature, still exhibited a peak temperature lower than the 0 % concrete. This indicates that in this test, the differences in initial temperatures and minor variations in ambient temperatures were insufficient to affect the influence of GGBS content on peak temperatures. However, they significantly impacted the time to reach peak temperature. The introduced new indicators “Temperature rise value (T_r_)” incorporate the impact of the initial temperature of the specimens and the ambient temperature during the curing process, thereby providing a more comprehensive understanding of the concrete's thermal behaviour.

**Table 6:** Summary of the concrete hydration temperature development.

	Initial temperature	Peak temperature	Time to reach peak temperature t_peak_ (h)	Temperature rise value
	T_ini_ (°C)	T_peak_ (°C)		T_r_ (°C)
0 % GGBS	17.85	29.65	15.40	11.80
30 % GGBS	20.05	26.10	15.25	6.05
50 % GGBS	19.00	23.05	17.30	4.05

### 4.2 Isothermal calorimetry test results

**Fig. 6:**
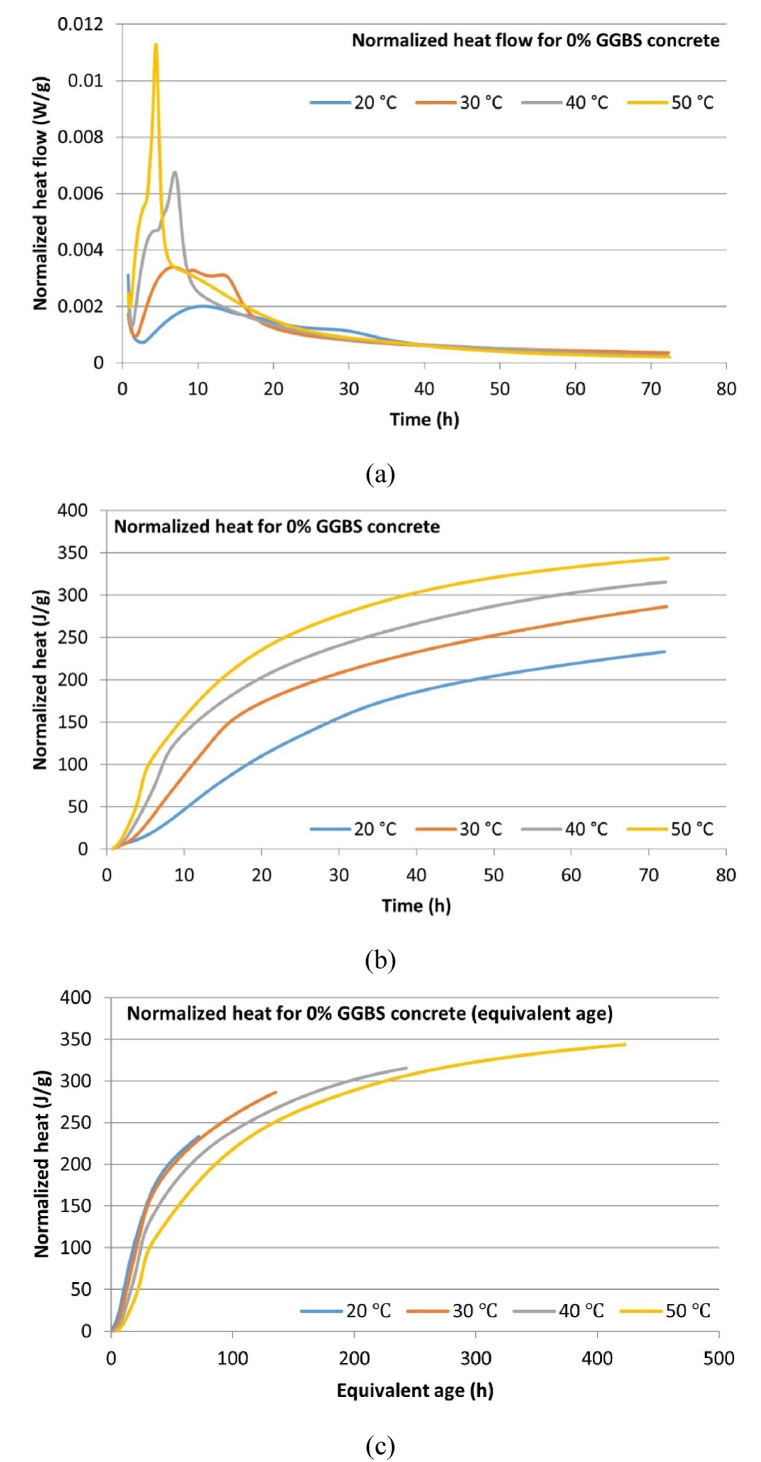
Hydration heat development of CEM-only (0 % GGBS) concrete: (a) Normalized heat flow; (b) Normalized cumulative heat; (c) Normalized cumulative heat (equivalent age).

**Fig. 7:**
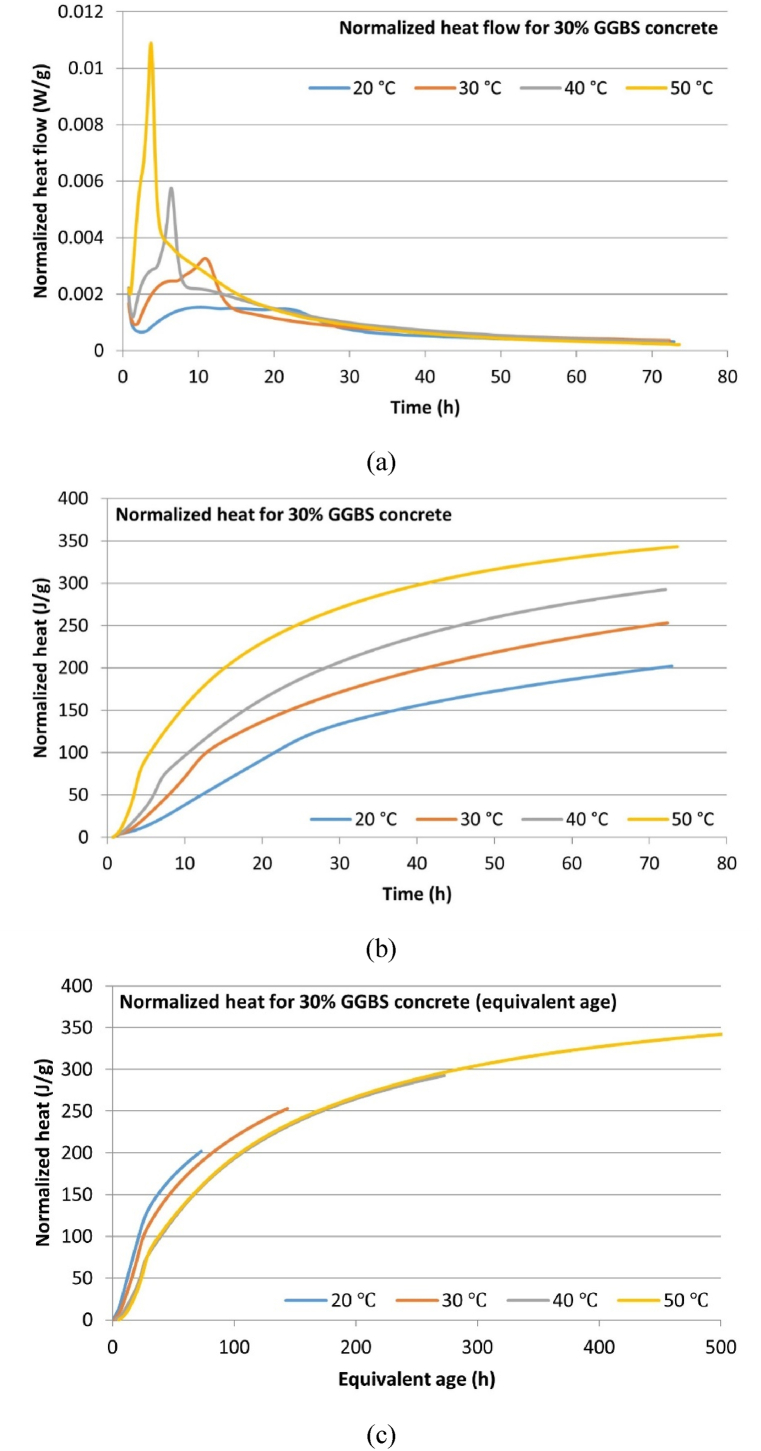
Hydration heat development of 30 % GGBS concrete: (a) Normalized heat flow; (b) Normalized cumulative heat; (c) Normalized cumulative heat (equivalent age).

**Fig. 8:**
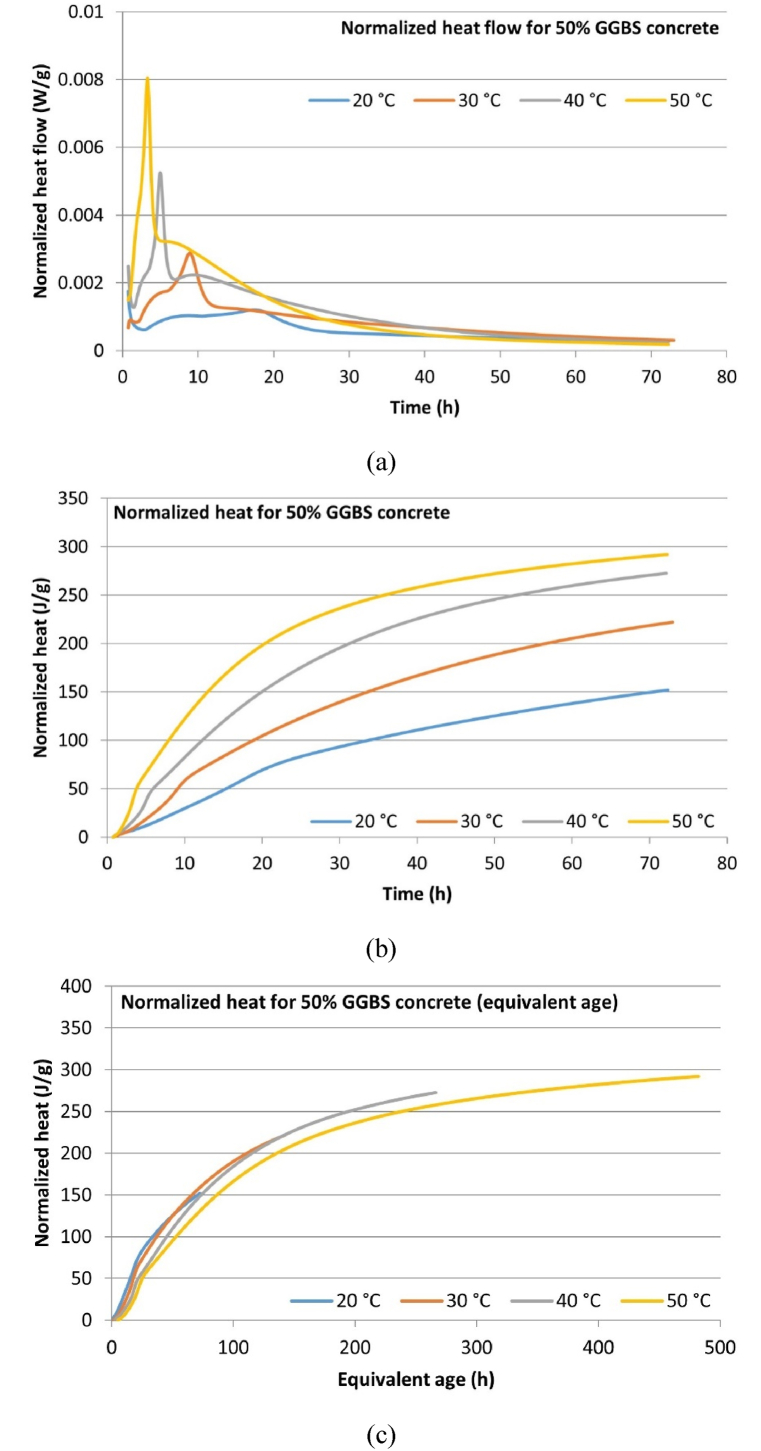
Hydration heat development of 50 % GGBS concrete: (a) Normalized heat flow; (b) Normalized cumulative heat; (c) Normalized cumulative heat (equivalent age).

The isothermal calorimetry normalized heat flow data and normalized cumulative hydration heat curves for the micro-concrete samples at different temperatures are depicted in Fig. 6, Fig. 7, Fig. 8. The normalization is based on the total mass of cementitious material (9 g), which includes both CEM I and GGBS. Table 7 summarizes the key hydration heat characteristics, including peak hydration heat, time to reach peak hydration, and 3-day cumulative hydration heat. The results indicate that for all mix samples, increasing the temperature leads to higher peak hydration heat values and 3-day cumulative hydration heat, while the time to reach peak hydration heat decreases. Additionally, replacing part CEM I with GGBS results in a reduction of peak hydration heat and 3-day cumulative hydration heat as the GGBS content increases, and the time to reach peak hydration heat extends accordingly. These findings indicate that elevated curing temperatures accelerate the hydration process, whereas incorporating GGBS as a replacement for CEM suppresses the generation of hydration heat. This suppression is primarily attributed to the delayed hydration of GGBS, which does not commence immediately but begins only after sufficient calcium hydroxide is produced by the hydration of CEM, creating an adequately alkaline environment for GGBS hydration to proceed.

**Table 7:** Hydration heat developments characteristics of micro-concrete samples.

Concrete mix	Curing temperature	Hydration peak value (W/g)	Time to reach peak (h)	3-day cumulative hydration heat (J/g)	Equivalent age t_e_ (h) at 72 h (3 days)
0 % GGBS concrete	20 °C	0.0020	10.69	225.97	72.00
	30 °C	0.0034	6.74	282.02	134.69
	40 °C	0.0068	6.95	312.12	242.07
	50 °C	0.0113	4.43	343.27	419.58
30 % GGBS concrete	20 °C	0.0015	10.30	195.59	72.00
	30 °C	0.0033	10.89	248.99	143.13
	40 °C	0.0057	6.41	289.30	272.32
	50 °C	0.0109	3.73	341.78	497.88
50 % GGBS concrete	20 °C	0.0012	17.50	145.54	72.00
	30 °C	0.0029	8.90	217.88	141.32
	40 °C	0.0052	4.97	268.37	265.69
	50 °C	0.0080	3.29	291.77	480.36

Based on the results of the isothermal calorimetry tests (Fig. 6, Fig. 7, Fig. 8), this study plotted the cumulative hydration heat development curves using equivalent age (t_e_) as the time unit, with 20 °C as the reference temperature, according to the equivalent age equation (Eq. (2)). The results are presented in Fig. 6, Fig. 7, Fig. 8.

The fundamental assumption of equivalent age is that concrete with the same mix and equivalent age will have the same strength and cumulative hydration heat development, regardless of its temperature history. The last column of Table 7 also summarizes the t_e_ values relative to 20 °C for samples cured for 72 h at different temperatures. It can be observed that elevated temperatures significantly increase the equivalent age of concrete. At 30 °C, 0 %, 30 %, and 50 % GGBS concrete correspond to 134.69, 143.13, and 141.32 h at 20 °C, respectively. The highest t_e_ is observed in 30 % GGBS concrete, reaching 497.88 h. This significant difference results from the definition of Eq. (2), where elevated temperatures cause an exponential increase in t_e_.

Isothermal calorimetry tests were conducted simultaneously on micro-concrete and equivalent mortar samples to evaluate the validity of using mortar hydration heat as a substitute for concrete. Given the extensive data obtained from these experiments, this section will focus on the results where significant differences in hydration heat development between micro-concrete and equivalent mortar samples were observed.

The test results indicate a significant overlap in the hydration heat flow curves between the micro-concrete and equivalent mortar samples. This high degree of similarity suggests that the hydration heat development of concrete and mortar samples is quite comparable. However, the primary distinction between micro-concrete and equivalent mortar samples lies in their 3-d cumulative hydration heat.

**Fig. 9:**
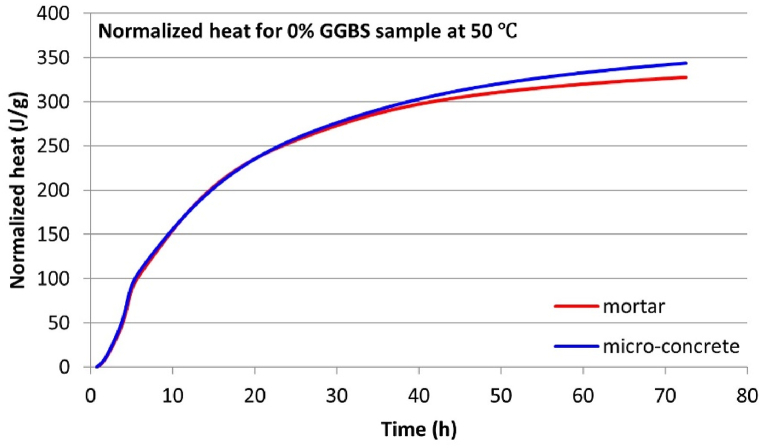
3-d cumulative hydration heat for 0 % GGBS samples at 50 °C.

**Fig. 10:**
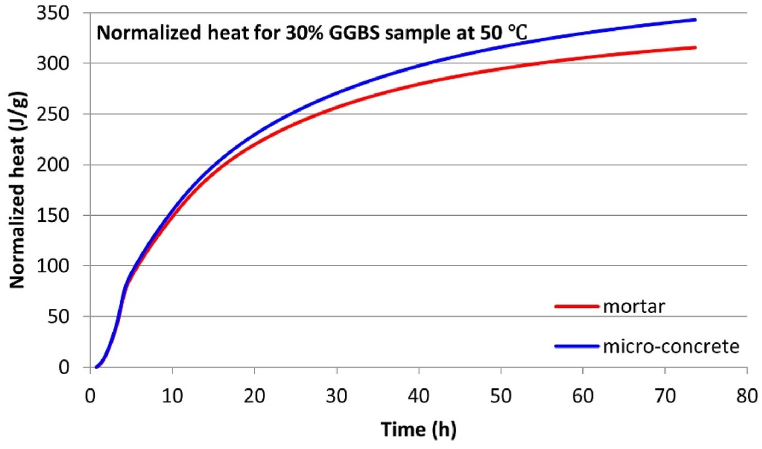
3-d cumulative hydration heat for 30 % GGBS samples at 50 °C.

**Fig. 11:**
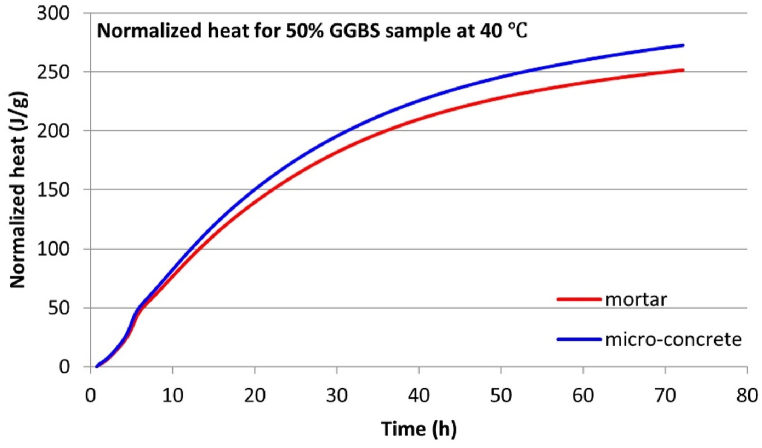
3-d cumulative hydration heat for 50 % GGBS samples at 40 °C.

One clear trend can be discerned: for all micro-concrete samples at temperatures equal to or greater than 30 °C, the 3-day cumulative hydration heat is consistently higher than that of their corresponding equivalent mortar samples. This suggests that including coarse aggregate enhances the development of hydration heat, an effect that accumulates over time and becomes distinctly evident after three days. Table 8 summarizes the 3-day cumulative hydration heat data to compare the hydration heat differences between these two sample types. The “Difference” column in Table 8 represents the 3-day cumulative hydration heat disparity between the micro-concrete samples and their corresponding equivalent mortar samples for each mix. It is consistently observed that this value is positive, indicating that the 3-day cumulative hydration heat for micro-concrete samples is invariably higher than that for the equivalent mortar samples when the curing temperature is at or above 30 °C. One notable observation is that the more significant “Difference” for each GGBS content mix typically occurs under higher temperature curing conditions (the maximum “Difference” for the 0 % and 30 % GGBS mixes occurs at 50 °C, while 50 % GGBS occurs at 40 °C, as shown in Fig. 9, Fig. 10, Fig. 11, separately. This study only displayed the curves with the largest “Difference” for each mix due to the space constraints. The rest of the experimental results are summarized in Table 8.). This may be attributed to the higher thermal conductivity of the solid coarse aggregates compared to the semi-fluid state of fresh mortar, with higher temperatures amplifying the disparity in heat conduction).

**Table 8:** Normalized 3-day cumulative hydration heat.

Sample information		Normalized 3-day cumulative hydration heat (J/g)		
GGBS content	Temperature	Mortar sample	Micro-concrete sample	Difference^a^
0 % GGBS	30 °C	274.90	282.02	7.12
	40 °C	310.03	312.12	2.09
	50 °C	327.38	343.27	15.89
30 % GGBS	30 °C	243.58	248.99	5.41
	40 °C	287.71	289.30	1.59
	50 °C	314.60	341.78	27.18
50 % GGBS	30 °C	213.86	217.88	4.02
	40 °C	248.58	268.37	19.79
	50 °C	283.54	291.77	8.23

a All differences in 3-day cumulative hydration heat values in this Table are Micro-concrete samples minus Mortar samples.

This research finding suggests that the role of coarse aggregates in the thermal transfer within concrete is non-negligible, especially for predicting the hydration heat development and thermal cracking in mass concrete using isothermal calorimetry. In mass concrete, the potential for reaching high hydration temperatures, possibly exceeding the maximum temperature of 50 °C applied in this study. As the hydration temperature increases, the “Difference” noted in Table 8 may be amplified and potentially leading to prediction inaccuracies.

### 4.3 Apparent activation energy and hydration parameters

The isothermal calorimetry results were employed for linear fitting (Eq. (3)) to obtain the apparent activation energy (E_a_) for micro-concrete and equivalent mortar samples with varying GGBS contents. The results are presented in Table 9. The “Differences” column in Table 9 represents the values for the micro-concrete minus mortar sample. The results indicate that the E_a_ values for all concrete mixes are slightly higher than those for mortar, with the difference appearing to increase with GGBS content. However, it is crucial to note that these differences are small (less than 2.5 %) and may fall within the range of experimental error. As observed in Section 4.2, significant differences in cumulative heat between micro-concrete and mortar only become apparent after approximately three days of hydration. In contrast, the Q_max_ values used to derive E_a_ typically occur within the first 20 h, where the differences between micro-concrete and mortar are less pronounced (less than 2.5 %). Therefore, while these results suggest a potential trend towards higher temperature sensitivity in micro-concrete compared to mortar, particularly with increasing GGBS content, the results cannot conclusively state that these differences are significant. Further investigation using complementary techniques such as XRD and TGA would be necessary to validate these findings and explore the underlying mechanisms.

**Table 9:** Apparent activation energy (E_a_).

Sample information	Apparent activation energy (J/mol)		
	Mortar sample	Micro-concrete sample	Difference^a^
0 % GGBS	46328.10	46373.23	45.13
30 % GGBS	49850.74	50765.28	914.54
50 % GGBS	48554.59	49824.97	1270.38

a All differences in E_a_ values in this Table are Micro-concrete samples minus Mortar samples.

Some literatures [51,52] suggest that the E_a_ value of concrete increases linearly with the percentage of GGBS replacing cement. However, in this study, the calculated activation energy for the 50 % GGBS mix is lower than that for the 30 % GGBS mix, which contradicts this conclusion. Several factors might contribute to this discrepancy. Firstly, differences in the chemical composition and physical properties of the cement and GGBS used in different studies can significantly impact hydration kinetics, thereby affecting the calculation of activation energy. Secondly, variations in the water-to-binder ratio across studies can play a crucial role in regulating the hydration process and its energy requirements.

**Fig. 12:**
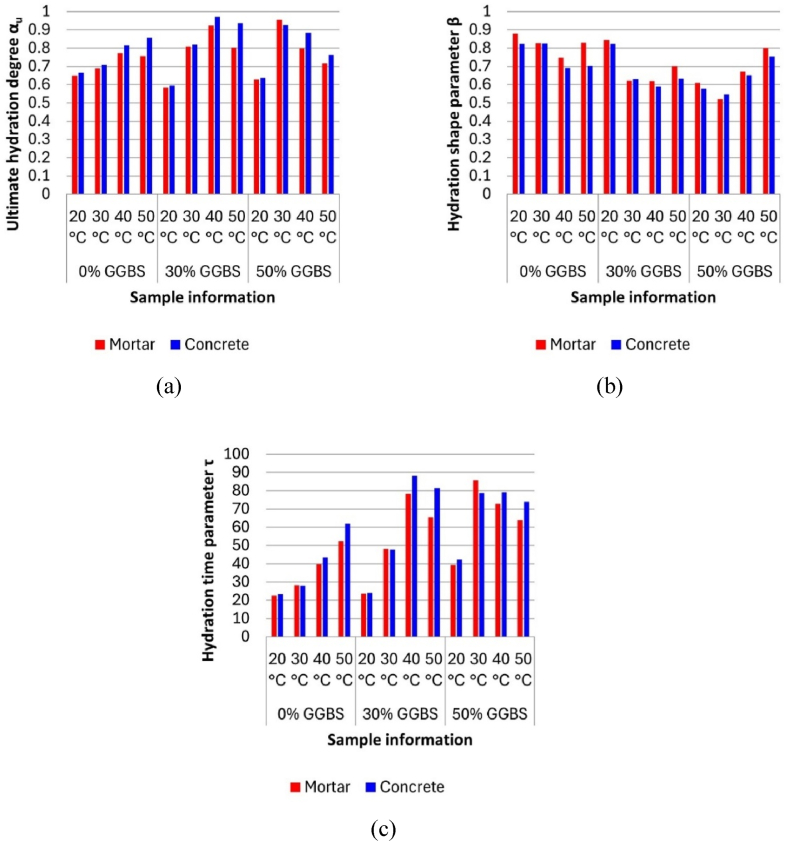
Hydration parameters fitting results: (a) α_u_; (b) β; (c) τ.

The hydration parameters for all samples were obtained through nonlinear fitting (Eq. (14)) of the isothermal calorimetry results, which are presented in Table 10. Fig. 12 illustrates the differences in the hydration parameter between concrete and mortar specimens via bar charts. The results indicate that the ultimate hydration degree (α_u_) for most concrete samples is higher than that of equivalent mortar. Similarly, the hydration time parameter (τ) for most concrete samples exceeds that of equivalent mortar. Furthermore, for all mixes, the differences in α_u_ and τ between concrete and mortar increase under high-temperature conditions. This indicates that concrete, in comparison to equivalent mortar, displays a greater ultimate hydration degree and a higher hydration delay, with these phenomena being more pronounced under elevated curing temperatures. However, the hydration shape parameter (β) for most concrete samples is lower than that of equivalent mortar. This indicate that concrete will have a lower hydration heat than equivalent mortar during early ages. The differences in hydration parameters between mortar and concrete due to the inclusion of coarse aggregates will be reflected in the development of hydration heat curves, potentially further impacting the accuracy of concrete temperature prediction results.

**Table 10:** Hydration parameters.

Sample information		Hydration parameters					
GGBS content	Temperature	α_u_		β		τ	
		Mortar	Concrete	Mortar	Concrete	Mortar	Concrete
0 % GGBS	20 °C	0.649	0.665	0.879	0.822	22.500	23.249
	30 °C	0.689	0.707	0.828	0.825	28.188	28.053
	40 °C	0.773	0.815	0.747	0.690	39.629	43.534
	50 °C	0.755	0.857	0.830	0.704	52.357	61.871
30 % GGBS	20 °C	0.583	0.594	0.844	0.823	23.599	23.933
	30 °C	0.809	0.818	0.621	0.629	48.065	47.708
	40 °C	0.924	0.972	0.620	0.591	78.268	88.181
	50 °C	0.803	0.936	0.701	0.631	65.609	81.408
50 % GGBS	20 °C	0.629	0.637	0.608	0.577	39.365	42.221
	30 °C	0.956	0.927	0.520	0.545	85.606	78.830
	40 °C	0.797	0.885	0.672	0.651	72.889	79.215
	50 °C	0.716	0.763	0.799	0.753	63.889	73.812

### 4.4 FEM modelling results

**Fig. 13:**
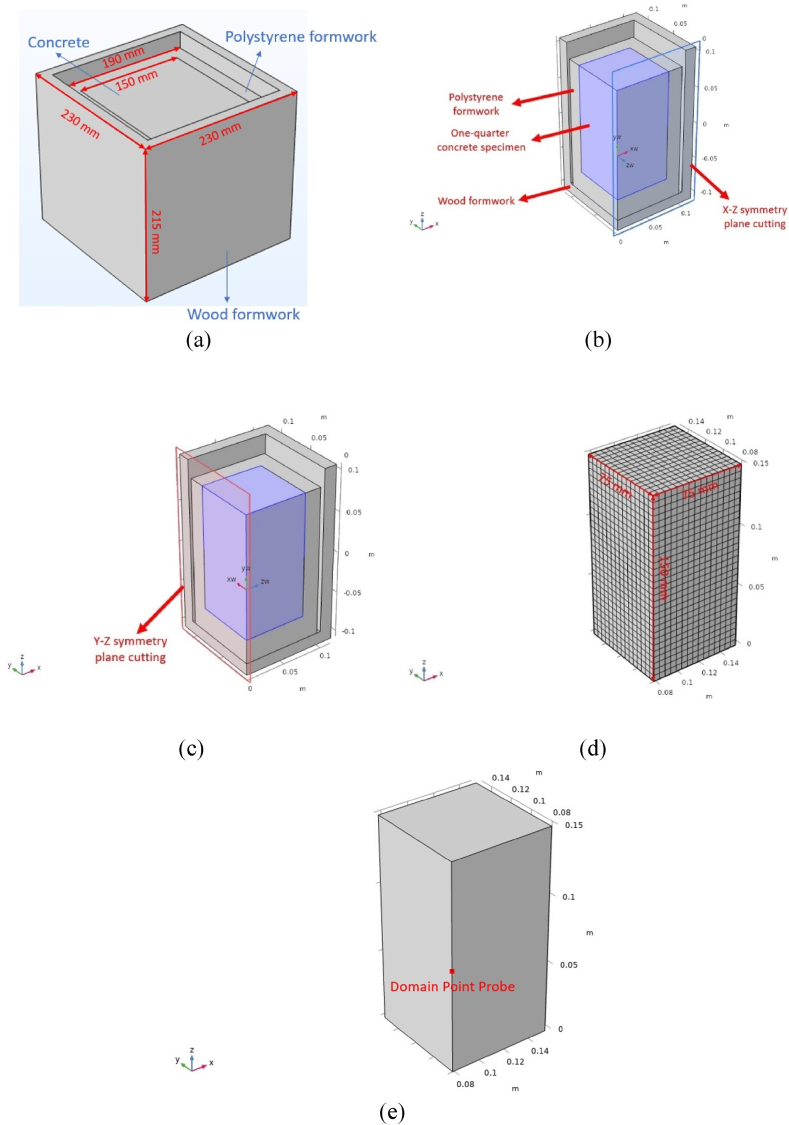
Concrete structural element modelling and one-quarter symmetric model: (a) Geometrical modelling of semi-adiabatic concrete specimen; (b) X-Z symmetry plane cutting; (c) X-Z symmetry plane cutting; (d) Concrete one-quarter symmetric model mesh results (without mould); (e) Extracted temperature points.

The developed FEM model is designed to simulate the temperature development of concrete specimens from semi-adiabatic calorimetry tests, thereby necessitating that the structural elements within the model mirror those of the concrete specimens utilized in the experiments. The initial geometric model created in COMSOL (concrete specimen with insulation layers) is shown in Fig. 13 (a). To optimize computational efficiency, the model leverages the symmetry planes in the XZ and YZ directions, dividing the model into four identical parts by cutting along these planes and using only one-quarter for calculations. Fig. 13 (b) and (c) respectively present the quarter-model schematics cut along the X-Z and Y-Z symmetry planes. This approach reduces the computational domain, significantly lowering the computational load while maintaining solution accuracy. Given the regular cubic geometry of the concrete specimen, a hexahedral meshing technique was employed. This method is selected for its efficiency in handling simple geometries and its ability to generate structured meshes, which are advantageous for the numerical stability and accuracy of finite element analysis [45]. The resulting mesh comprised 6525 elements. Fig 13 (d) shows the meshed quarter-symmetry concrete specimen. The temperature evolution at the centre of the concrete specimen was monitored via COMSOL's built-in Domain Point Probe feature. In this one-quarter symmetric model, the centre point of the concrete specimen is represented by the midpoint of the intersection line between the two symmetry planes of the original geometric model, as illustrated in Fig. 13 (e).

**Fig. 14:**
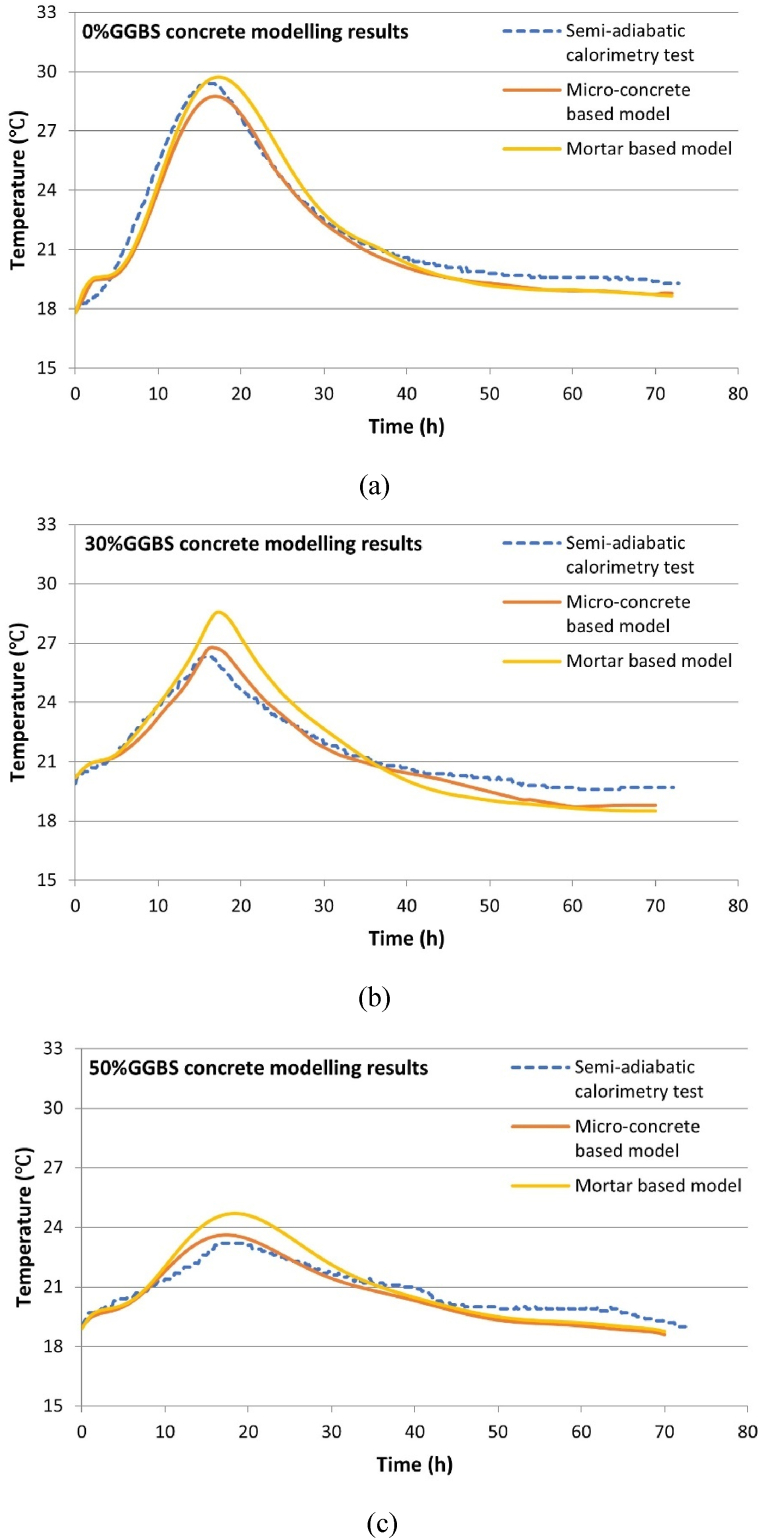
Concrete temperature FEM modelling results.

The boundary conditions used in this FEM model are derived from the ambient temperatures monitored during the semi-adiabatic calorimetry tests. These ambient temperatures, shown in Fig. 5, fluctuate and directly influence the concrete's heat loss through thermal convection. This setup ensures that the FEM model accurately reflects the actual conditions experienced by the concrete specimens during the tests. Fig. 14 presents the simulation results of the concrete temperature FEM model. The blue dashed line represents the semi-adiabatic calorimetry test results, which serve as a benchmark for validating the simulation accuracy. The orange curve indicates the model's heat source derived from the isothermal calorimetry tests of the micro-concrete sample, termed the “concrete model”. The yellow curve represents the model's heat source derived from the isothermal calorimetry tests of the equivalent mortar sample, termed the “mortar model”. It can be observed that, for all three mixes, the accuracy of the concrete model surpasses that of the mortar model. In the simulation results of the 0 % GGBS concrete, although the peak temperature error of the concrete model is slightly larger than that of the mortar model, the overall accuracy of the mortar model curve is lower than that of the concrete model, particularly during the cooling phase from 15 to 30 h. For the 30 % and 50 % GGBS concrete, the mortar model's accuracy is evidently lower than that of the concrete model, especially concerning the peak temperature and the cooling phase.

Table 11, Table 12 summarize the simulation errors for the peak temperature and the time to reach the peak temperature, respectively. Almost all concrete models exhibit higher accuracy compared to mortar models, with the only exception of the 0 % GGBS concrete's peak temperature, where the concrete model's error is 3 %, higher than the mortar model's 0.24 %. However, 3 % is still within an acceptable error range (generally less than 5 %). In contrast, the mortar model's simulation results show multiple errors exceeding 5 %, specifically for the peak temperatures of the 30 % and 50 % GGBS concrete and the time to peak temperature for the 0 % and 30 % GGBS concrete.

**Table 11:** Peak hydration temperature modelling results.

GGBS content	Concrete peak temperature (°C) modelling results and errors				
	Test	Concrete model	Concrete model error	Mortar model	Mortar model error
0 %	29.65	28.76	3.00 %	29.72	0.24 %
30 %	26.10	26.75	2.49 %	28.54	9.35 %
50 %	23.05	23.61	2.43 %	24.69	7.11 %

**Table 12:** Time to reach peak temperature modelling results.

GGBS content	Time to reach peak temperature (h) modelling results and errors				
	Test	Concrete model	Concrete model error	Mortar model	Mortar model error
0 %	15.40	16.00	3.90 %	16.60	7.79 %
30 %	15.25	15.90	4.26 %	16.50	8.19 %
50 %	17.30	17.20	1.19 %	17.80	2.89 %

Notably, during the heating phase for all samples, the mortar model curves are generally steeper (indicating a higher average slope), leading these mortar models to reach higher peak temperatures. This pattern aligns with theoretical expectations because the heat source part of the concrete temperature FEM modelling is based on the TPE equation (Eq. (14)), which includes three hydration parameters that determine the shape and progress of the hydration heat curve. Although GGBS content and hydration temperature may influence the hydration parameters, almost all concrete samples exhibit higher hydration time parameters (τ) and lower hydration shape parameters (β) than the mortar samples. These indicate that the presence of coarse aggregates in concrete delays the reaction and reduces the early-age hydration rate separately, explaining why the mortar model's temperature rise curve is steeper during the heating phase than that of the concrete model. This observation directly corroborates and supports the hypothesis proposed by Xu et al. [37] that the delaying effect of coarse aggregates could lead to errors in concrete temperature simulation results based on the mortar isothermal calorimetry tests.

## 5 Conclusion

This study employed semi-adiabatic and isothermal calorimetry tests to evaluate the effects of replacing portions of CEM I with GGBS on the hydration temperature and heat development in concrete. Isothermal calorimetry tests at various temperatures measured the hydration heat development of concrete and equivalent mortar samples with different GGBS contents to explore the influence of coarse aggregates on cement hydration heat. Concrete temperature FEM models were established based on hydration data from both concrete and equivalent mortar to investigate potential errors in temperature predictions caused by using mortar hydration data. The accuracy of the models was validated against temperature results from semi-adiabatic calorimetry tests. The main conclusions are as follows:

1. Concrete temperature and hydration heat development: Semi-adiabatic calorimetry tests indicated that replacing CEM I with GGBS significantly reduces the peak temperature and delays the time to reach peak temperature in concrete. Results from isothermal calorimetry showed that higher curing temperatures promote hydration heat development, whereas GGBS substitution inhibits it. These test results highlight the effectiveness of GGBS in mitigating concrete early-age hydration heat and thermal cracking risks.
2. Role of Coarse Aggregates: Isothermal calorimetry tests demonstrated that the inclusion of coarse aggregates increases hydration heat development, particularly at elevated curing temperatures. This can be attributed to the higher thermal conductivity of coarse aggregates compared to the semi-fluid state of fresh mortar. As a result, micro-concrete transfers more hydration heat to the calorimeter than mortar samples. This effect becomes more pronounced with increasing curing temperatures.
3. Apparent activation energy: The apparent activation energy (E_a_) derived from isothermal calorimetry results of micro-concrete samples were generally higher than those from equivalent mortar samples. However, these differences were small (less than 2.5 %) and may be attributable to experimental variation. While this might suggest a trend towards greater temperature sensitivity in the presence of coarse aggregates, especially with the addition of GGBS, further research employing complementary analytical techniques is needed to draw definitive conclusions about the temperature sensitivity differences between mortar and concrete.
4. Hydration Parameters: Most micro-concrete samples exhibited higher ultimate hydration degree (α_u_) and hydration time parameter (τ) but lower hydration shape parameter (β) compared to equivalent mortar samples. These differences in hydration parameters suggest that concrete samples have greater ultimate hydration heat, longer higher hydration delay, and lower early hydration rates than mortar samples. This indicates that the absence of coarse aggregates in mortar samples can lead to discrepancies in hydration heat development compared to concrete.
5. FEM model results showed that temperature predictions using hydration data from micro-concrete samples were more accurate than those using data from mortar samples. This is primarily due to the hydration parameters derived from isothermal calorimetry data being incorporated into the FEM model's heat source. This finding highlights the errors in concrete temperature predictions when the effect of coarse aggregates is neglected.

In conclusion, this study underscores the indispensable role of coarse aggregates in cement hydration heat development. For concrete temperature prediction models based on calorimetry tests, this study recommended employing micro-concrete samples that fully replicate the actual concrete mix, rather than mortar samples, to accurately capture the hydration heat development of concrete.

## Ethics approval and consent to participate

Not applicable.

## Data availability statement

Data will be made available on request.

## CRediT authorship contribution statement

**Yaowen Tan:** Writing – review & editing, Writing – original draft, Visualization, Validation, Software, Resources, Methodology, Investigation, Formal analysis, Data curation, Conceptualization. **Kangkang Tang:** Writing – review & editing, Supervision, Resources, Project administration, Methodology, Investigation, Conceptualization.

## Declaration of competing interest

The authors declare that they have no known competing financial interests or personal relationships that could have appeared to influence the work reported in this paper.
